# Electrochemical Oscillation during Galvanostatic Charging of LiCrTiO_4_ in Li-Ion Batteries

**DOI:** 10.3390/ma14133624

**Published:** 2021-06-29

**Authors:** Zhijie Xu, Fangxu Hu, De Li, Yong Chen

**Affiliations:** 1 State Key Laboratory on Marine Resource Utilization in South China Sea, Hainan Provincial Key Laboratory of Research on Utilization of Si-Zr-Ti Resources, School of Materials Science and Engineering, Hainan University, Haikou 570228, China; 18085204210049@hainanu.edu.cn (Z.X.); 19085204210019@hainu.edu.cn (F.H.); 2 Guangdong Key Laboratory for Hydrogen Energy Technologies, School of Materials Science and Hydrogen Energy, Foshan University, Foshan 528000, China

**Keywords:** Li-ion battery, LiCrTiO_4_, electrochemical oscillation, phase transition, spinel structure

## Abstract

In the late 1960s, the establishment of Prigogine’s dissipative structure theory laid the foundation for the (electro)chemical oscillation phenomenon, which has been widely investigated in some electrochemical reactions, such as electro-catalysis and electro-deposition, while the electrochemical oscillation of Li-ion batteries has just been discovered in spinel Li_4_Ti_5_O_12_ a few years before. In this work, spinel LiCrTiO_4_ samples were synthesized by using a high-temperature solid-state method, characterized with SEM (Scanning electron microscope), XRD (X-ray diffraction), Raman and XPS (X-ray photoelectron spectroscopy) measurements, and electrochemically tested in Li-ion batteries to study the electrochemical oscillation. When sintering in a powder form at a temperature between 800 and 900 °C, we achieved the electrochemical oscillation of spinel LiCrTiO_4_ during charging, and it is suppressed in the non-stoichiometric LiCrTiO_4_ samples, especially for reducing the Li content or increasing the Cr content. Therefore, this work developed another two-phase material as the powder-sintered LiCrTiO_4_ exhibiting the electrochemical oscillation in Li-ion batteries, which would inspire us to explore more two-phase electrode materials in Li-ion batteries, Na-ion batteries, etc.

## 1. Introduction

Li-ion batteries have been widely used in portable electronic devices and electric vehicles, [1,2,3] and graphite is a commercial anode material in Li-ion batteries, while it suffers from poor rate capability and serious safety issues, owing to lithium dendritic growth, especially at low temperature [4,5]. Spinel Li_4_Ti_5_O_12_ is considered among the promising anode materials, which can prevent the growth of lithium dendrites to achieve safe and reliable high-power Li-ion batteries, owing to the high operating voltage of 1.565 V [6,7,8,9]. Since Li_4_Ti_5_O_12_ has smooth discharge and charge plateaus, even subtle voltage changes can be observed on the discharge/charge curves, such as the memory effect and the electrochemical oscillation [10,11,12,13].

In the late 1960s, the establishment of Prigogine’s dissipative structure theory laid the foundation for the (electro)chemical oscillation phenomenon, which attracted a lot of attention in some electrochemical reactions, such as electro-synthesis [14,15], electrocatalysis [16,17] and electrodeposition [18,19]. The electrochemical oscillation can not only be adopted to interpret some interesting electrochemical phenomena, but also be developed for some potential applications [20,21]. However, the electrochemical oscillation of Li-ion batteries has just been discovered in spinel Li_4_Ti_5_O_12_ a few years before, which presents as a periodic voltage oscillation during the galvanostatic process in Li-ion batteries. Up to now, there is no report about the electrochemical oscillation of any other two-phase materials in Li-ion batteries.

Recently, LiCrTiO_4_ has been comprehensively studied due to its similar characteristics to Li_4_Ti_5_O_12_ [22,23,24,25]. In this work, spinel LiCrTiO_4_ was synthesized by using a high-temperature solid-state method, characterized with SEM, XRD, Raman and XPS measurements, and electrochemically tested in Li-ion batteries. Through tailoring the sintering temperature, the elemental ratio of Li:Cr:Ti, and the sintering form as powder or pellet, we found that the electrochemical oscillation can be achieved in the stoichiometric LiCrTiO_4_ sintered in a powder form between 800 and 900 °C. This work firstly discovered the electrochemical oscillation in another two-phase material of spinel LiCrTiO_4_, which can promote the investigation of electrochemical oscillation in Li-ion batteries, Na-ion batteries, etc.

## 2. Experimental Section

Spinel LiCrTiO_4_ was synthesized by using a high-temperature solid-state method. The raw materials as Li_2_CO_3_ (99.9%, Xilong Chemical, Guangzhou, China), Cr_2_O_3_ (99.9%, Aladdin, Shanghai, China) and TiO_2_ (nano-sized, 99.9%, Aladdin, Shanghai, China) were weighted with an elemental ratio of Li:Cr:Ti as 1:1:1, their mixture was thoroughly ground for 2 h, then the powder was directly sintered in a muffle furnace with air atmosphere at a temperature of 750, 800, 850, 900 and 950 ℃, respectively, for 16 h, in which the product of 850 ℃ was marked as LiCrTiO_4_-850-powder [26]. In comparison, the ground powder was pressed into a pellet and sintered in a muffle furnace with air atmosphere at a temperature of 850 ℃ for 16 h, and the product was marked as LiCrTiO_4_-850-pellet. By using the same method, another six LiCrTiO_4_ samples were synthesized with an elemental ratio of Li:Cr:Ti as 1.05:1:1, 0.95:1:1, 1:1.05:1, 1:0.95:1, 1:1:1.05 and 1:1:0.95, respectively, which were sintered in a powder form at a temperature of 850 °C.

As-prepared LiCrTiO_4_ samples were characterized using an X-ray diffractometer (XRD, Bruker D8 advance, Bruker, Karlsruhe, Germany), a Raman spectrometer (Raman, Thermo Fisher DXRxi, Madison, WI, USA) using an argon laser with a wavelength of 532 nm, a scanning electron microscope (SEM, Phenom ProX, Phenom-World BV, Eindhoven, Netherlands), and an X-ray photoelectron spectroscope (XPS, ESCALAB 250xi, Thermo Fisher Scientific, Waltham, MA, USA) with a focused monochromatized Al-Ka radiation (1486.6 eV). Electrochemical measurements were conducted within coin-type cells (CR2025). The working electrode was a composite film (ϕ = 4 mm) firmly pressed on a carbon paper, which contained 42.5 wt.% active material, 42.5 wt.% acetylene black and 15 wt.% polytetrafluoroethylene (PTFE), the counter electrode of lithium metal was separated from the working electrode with a Celgard 2500 (Celgard, Charlotte, NC, USA) microporous polypropylene film, and the electrolyte was 1 M LiClO_4_/EC + DEC (volume ratio of 1:1). The dried components were assembled in a glovebox filled with Ar gas. The galvanostatic (dis)charging measurements were conducted at a current rate of 0.1 C between 1.2 and 2.0 V in the Hokuto Denko battery test system (HJ1001SD8, Hokuto Denko Corporation, Gifu, Japan) under an operating temperature of 25 °C.

## 3. Results and Discussion

**Figure 1:**
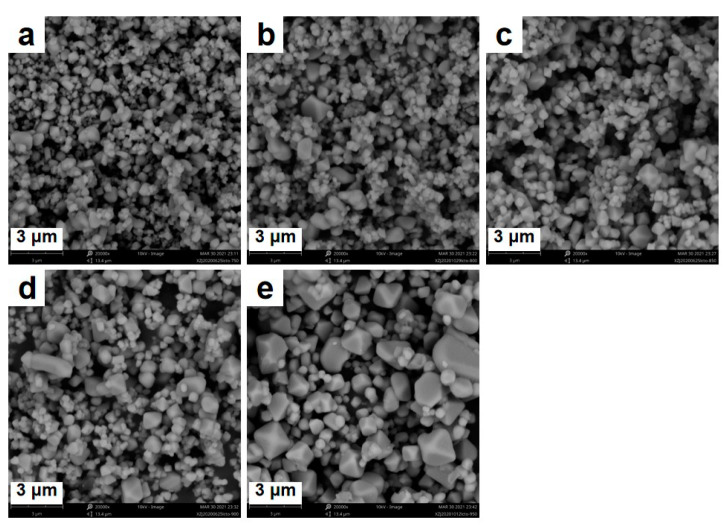
The SEM (Scanning electron microscope) images of LiCrTiO_4_ sintered in a powder form at a temperature of (**a**) 750 °C; (**b**) 800 °C; (**c**) 850 °C; (**d**) 900 °C and (**e**) 950 °C.

**Figure 2:**
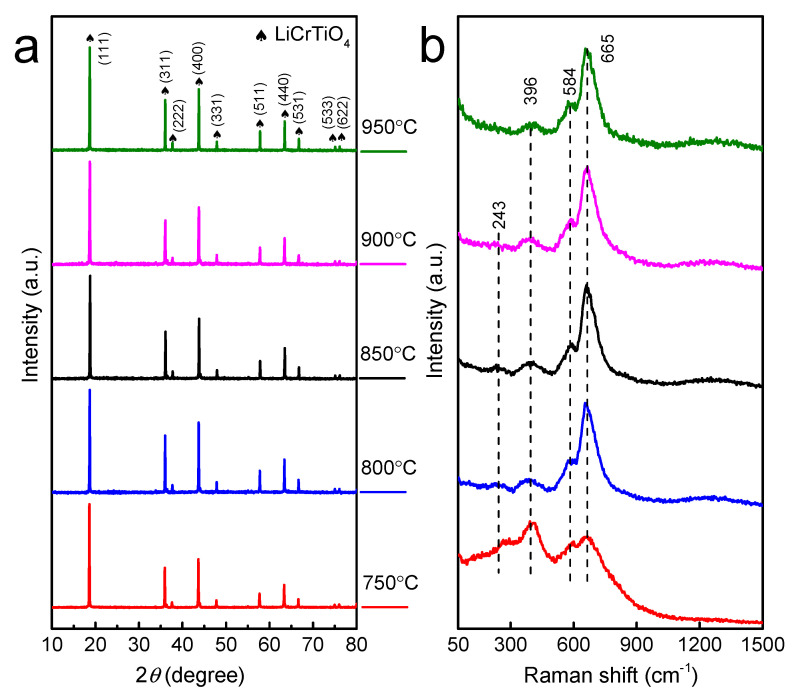
(**a**) The XRD (X-ray diffraction)patterns and (**b**) the Raman spectra of LiCrTiO_4_ sintered in a powder form at a temperature of 750 °C (red), 800 °C (blue), 850 °C (black), 900 °C (magenta) and 950 °C (olive).

Figure 1 shows the SEM images of LiCrTiO_4_ sintered in a powder form at a temperature of 750, 800, 850, 900 and 950 °C, respectively. Each sample is an aggregation of sub-microparticles and microparticles, and the particle size becomes large at the high temperature. As shown in Figure 2a and Figure S1, their XRD patterns are consistent with the standard Bragg reflections in JCPDS No. 47-0139, assigned to a spinel phase with space group Fd-3m [27,28]. There are four peaks at 243, 396, 584 and 665 cm^−1^ in their Raman spectra, as shown in Figure 2b. The peaks at 584 and 665 cm^−1^ can be attributed to the vibrational modes of Cr–O bonds in CrO_6_ octahedra and Ti–O bonds in TiO_6_ octahedra. The peak at 396 cm^−1^ can be assigned to the stretching vibrational mode of Li–O bonds in LiO_4_ tetrahedra. Additionally, the peak at 243 cm^−1^ is ascribed to the vibration of Li–O bonds [29,30]. For the LiCrTiO_4_ sintered at 750 ℃, the peak at 396 cm^−1^ is higher and the peak at 665 cm^−1^ is lower than that of high temperatures. Additionally, the peak at 243 cm^−1^ disappears at the high temperature of 900 and 950 °C. Accordingly, the local structure of LiCrTiO_4_ is sensitive to the sintering temperature, compared with its crystal structure.

**Figure 3:**
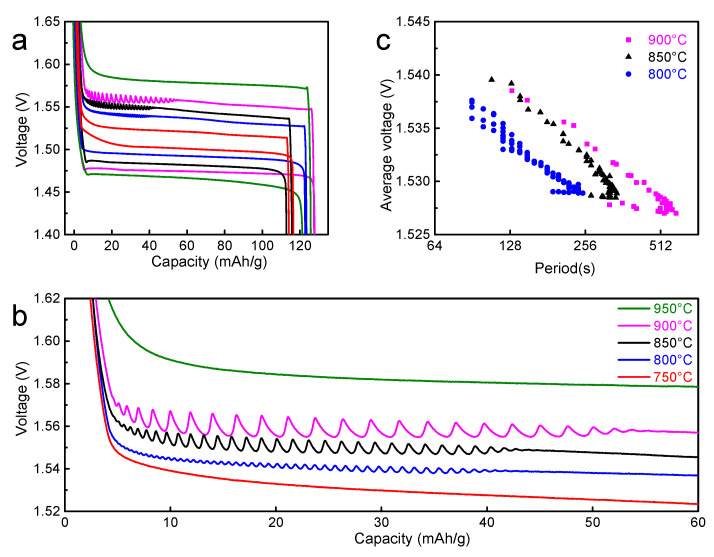
(**a**) The galvanostatic discharge/charge curves of LiCrTiO_4_ sintered in a powder form at a temperature of 750, 800, 850, 900 and 950 °C, respectively. (**b**) The enlarged view in the end of charge plateaus. The data are shifted along the vertical axis (750 °C ±0 mV; 800 °C ±10 mV; 850 °C ±20 mV; 900 °C ±30 mV; 950 °C ±40 mV) for viewing convenience. (**c**) The relationship between the period and the average voltage, which were calculated from (**b**).

The galvanostatic (dis)charging measurements were conducted for these LiCrTiO_4_ samples, as shown in Figure 3, where the current rate was 0.1 C and the operating temperature was 25 °C. As shown in Figure 3a,b, the charging curve is very smooth for the LiCrTiO_4_ sintered at 750 °C. The electrochemical oscillation (voltage oscillation) appears for the LiCrTiO_4_ sintered at 800 °C, and its range and amplitude increase continuously as the temperature rises from 800 to 900 °C. However, the electrochemical oscillation disappears completely when the sintering temperature reaches 950 °C. To analyze the oscillation, the relationship between the period and the average voltage was plotted in Figure 3c. At the beginning of electrochemical oscillation, the period gradually increases, while the average voltage is nearly constant. Then, the period and the average voltage gradually become smaller and higher, respectively, and the period basically has a negative logarithmic relationship with the average voltage. By increasing the temperature, the negative logarithmic lines gradually shift to the large period, possibly owing to the fact that the growing particle size prolongs the time of phase transition in each particle, as shown in Figure 1. Therefore, the electrochemical oscillation of LiCrTiO_4_ is significantly dependent on the sintering temperature, and the middle temperature of 850 °C was chosen in the following study, for this temperature is very popular to synthesize the spinel LiCrTiO_4_.

**Figure 4:**
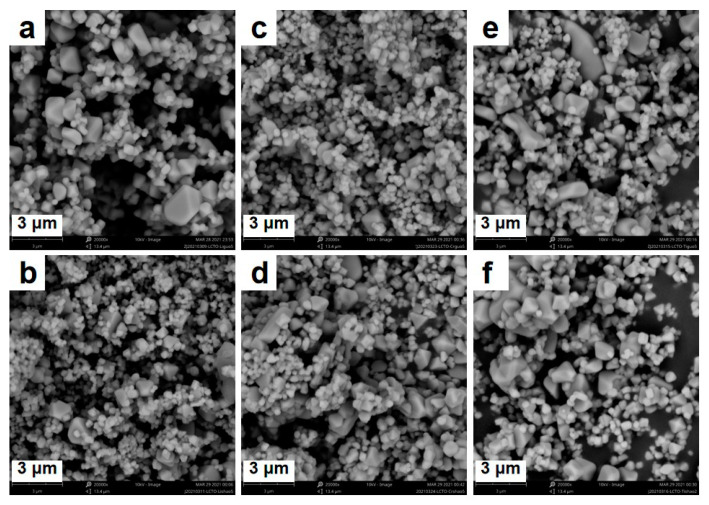
The SEM images of LiCrTiO_4_ sintered in a powder form at a temperature of 850 °C with an elemental ratio of Li:Cr:Ti as (**a**) 1.05:1:1; (**b**) 0.95:1:1; (**c**) 1:1.05:1; (**d**) 1:0.95:1; (**e**) 1:1:1.05 and (**f**) 1:1:0.95, respectively.

**Figure 5:**
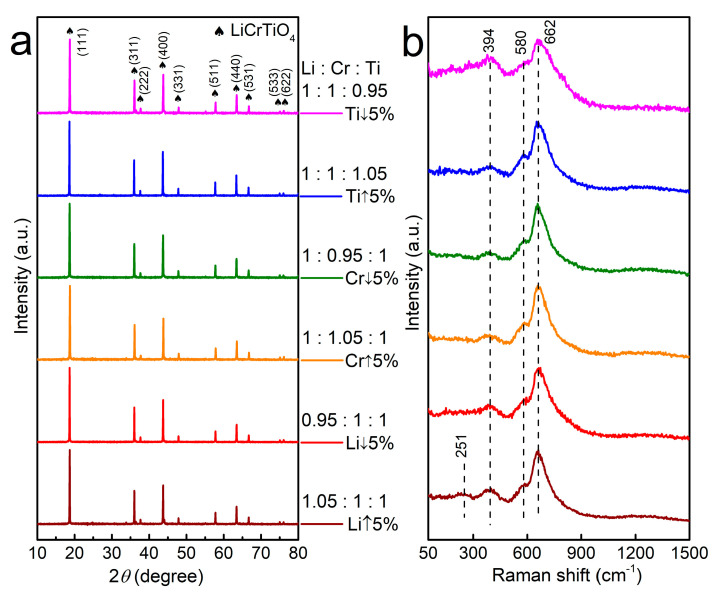
(**a**) The XRD patterns and (**b**) the Raman spectra of LiCrTiO_4_ sintered in a powder form at a temperature of 850 °C with an elemental ratio of Li:Cr:Ti as1.05:1:1 (wine), 0.95:1:1 (red), 1:1.05:1 (orange), 1:0.95 (olive):1, 1:1:1.05 (blue) and 1:1:0.95 (magenta), respectively.

The elemental ratio was tailored to study the electrochemical oscillation of LiCrTiO_4_. The elemental ratio of Li:Cr:Ti was controlled as 1.05:1:1, 0.95:1:1, 1:1.05:1, 1:0.95:1, 1:1:1.05 and 1:1:0.95 in a series of LiCrTiO_4_ samples, which were sintered in a powder form at a temperature of 850 °C. As shown in Figure 4, all sample appear as an aggregation of sub-micro-particles and micro-particles, and the particle size seems large by increasing the Li content (1.05:1:1 vs. 0.95:1:1) or reducing the Cr content (1:1.05:1 vs. 1:0.95:1), while there was no evident difference by varying the Ti content (1:1:1.05 vs. 1:1:0.95). As shown in Figure 5a and Figure S2, their XRD patterns are consistent with the spinel structure of LiCrTiO_4_. As shown in Figure 5b, similar Raman spectra were observed by varying the Li content (1.05:1:1 vs. 0.95:1:1) or the Cr content (1:1.05:1 vs. 1:0.95:1), while the peaks at 580 cm^−1^ and 662 cm^−1^ became broad by reducing the Ti content (1:1:1.05 vs. 1:1:0.95). Additionally, the peak at 251 cm^−1^ is only observed in the LiCrTiO_4_ of Li:Cr:Ti = 1.05:1:1. Thus, both the local structure and the crystal structure of LiCrTiO_4_ are quite robust under non-stoichiometry.

**Figure 6:**
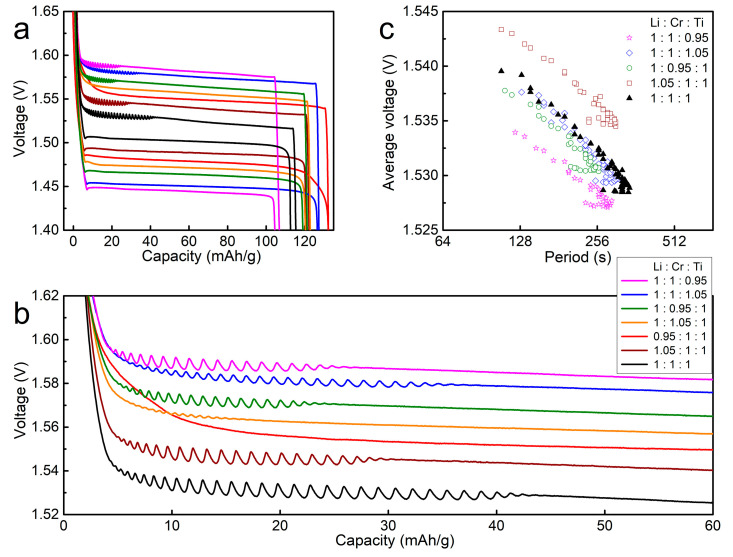
(**a**) The galvanostatic discharge/charge curves of LiCrTiO_4_ sintered in a powder form at a temperature of 850 °C with an elemental ratio of Li:Cr:Ti as 1:1:1 (black), 1.05:1:1 (wine), 0.95:1:1 (red), 1:1.05:1 (orange), 1:0.95:1 (olive), 1:1:1.05 (blue) and 1:1:0.95 (magenta), respectively. (**b**) The enlarged view in the end of charge plateaus. The data are shifted along the vertical axis (1:1:1 ±0 mV; 1.05:1:1 ±10 mV; 0.95:1:1 ±20 mV; 1:1.05:1 ±30 mV; 1:0.95:1 ±40 mV; 1:1:1.05 ±50 mV; 1:1:0.95 ±60 mV) for viewing convenience. (**c**) The relationship between the period and the average voltage, which were calculated from (**b**).

Figure 6 shows the galvanostatic discharge/charge curves of the LiCrTiO_4_ samples with different elemental ratios. Compared with the stoichiometric LiCrTiO_4_ (Li:Cr:Ti = 1:1:1), the electrochemical oscillation is reduced in range and amplitude for the non-stoichiometric LiCrTiO_4_ samples. As to each element, the electrochemical oscillation disappears by reducing the Li content (Li:Cr:Ti = 0.95:1:1) or increasing the Cr content (Li:Cr:Ti = 1:1.05:1), while it always exists by varying the Ti content (Li:Cr:Ti = 1:1:1.05 or 1:1:0.95). As shown in Figure 6c, the negative logarithmic line shifts to the high voltage for the LiCrTiO_4_ of Li:Cr:Ti = 1.05:1:1, and moves to the low voltage for the LiCrTiO_4_ of Li:Cr:Ti = 1:1:0.95. Thereby, the electrochemical oscillation strongly depends on the elemental ratio, especially for the Li and Cr contents.

**Figure 7:**
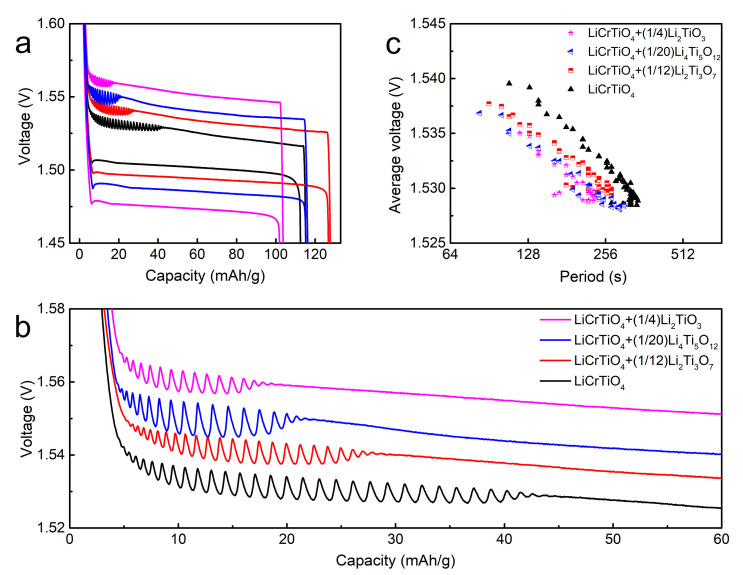
(**a**) The galvanostatic discharge/charge curves of LiCrTiO_4_ (black), the composite of LiCrTiO_4_ +$\;\frac{1}{12}\cdot$Li_2_Ti_3_O_7_ (red), the composite of LiCrTiO_4_ +$\;\frac{1}{20}\cdot$ Li_4_Ti_5_O_12_ (blue) and the composite of LiCrTiO_4_ +$\;\frac{1}{4}\cdot$ Li_2_TiO_3_ (magenta), which were sintered in a powder form at a temperature of 850 °C. (**b**) The enlarged view in the end of charge plateaus. The data are shifted along the vertical axis (LiCrTiO_4_ ±0 mV; LiCrTiO_4_ +$\;\frac{1}{12}\cdot$ Li_2_Ti_3_O_7_ ±10 mV; LiCrTiO_4_ + $\frac{1}{20}\cdot$ Li_4_Ti_5_O_12_ ±20 mV; LiCrTiO_4_ +$\;\frac{1}{4}\cdot$ Li_2_TiO_3_ ±30 mV) for viewing convenience. (**c**) The relationship between the period and the average voltage, which were calculated from (**b**).

Additionally, we also synthetized the composite of LiCrTiO_4_ + Li_2_Ti_3_O_7_, LiCrTiO_4_ + Li_4_Ti_5_O_12_ and LiCrTiO_4_ + Li_2_TiO_3_ to study the effect of different lithium titanates, as shown in Figure S3 [31]. As shown in Figure 7a,b, the range of electrochemical oscillation gradually becomes smaller as the Li content in lithium titanate increases, consistent with that of the LiCrTiO_4_ samples (Li:Cr:Ti = 1.05:1:1 vs.1:1:1) in Figure 6, and the amplitude of electrochemical oscillation is largest for the composite of LiCrTiO_4_ + Li_4_Ti_5_O_12_, indicating a positive effect of spinel Li_4_Ti_5_O_12_. As shown in Figure 7c, the negative logarithmic lines shift to the low voltage for the LiCrTiO_4_ composites. Thus, the electrochemical oscillation is affected by the Li content in lithium titanates, as well as spinel Li_4_Ti_5_O_12_ phase in the composite.

**Figure 8:**
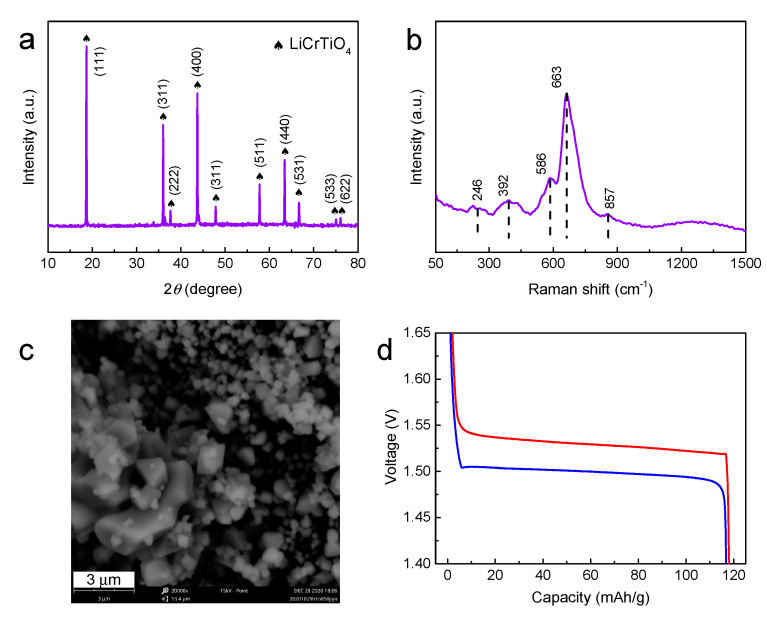
(**a**) The XRD pattern, (**b**) the Raman spectrum, (**c**) the SEM image and (**d**) the galvanostatic discharge/charge curves of LiCrTiO_4_ sintered in a pellet form at a temperature of 850 °C.

Usually, the raw materials are pressed into a pellet for sintering in the high-temperature solid-state method, so we also synthesized LiCrTiO_4_-850-pellet sintered in a pellet form at 850 °C. Compared with LiCrTiO_4_-850-powder, the LiCrTiO_4_-850-pellet has a similar XRD pattern and Raman spectrum, while there is an additional minor peak at 857 cm^−1^ which should originate from the stretching vibration of Cr^6+^=O as shown in Figure 8a,b, and the particles evidently become large in the LiCrTiO_4_-850 pellet, as shown in Figure 8c. The galvanostatic discharge/charge curves are plotted in Figure 8d, in which there is no electrochemical oscillation at the end of charging plateau.

**Figure 9:**
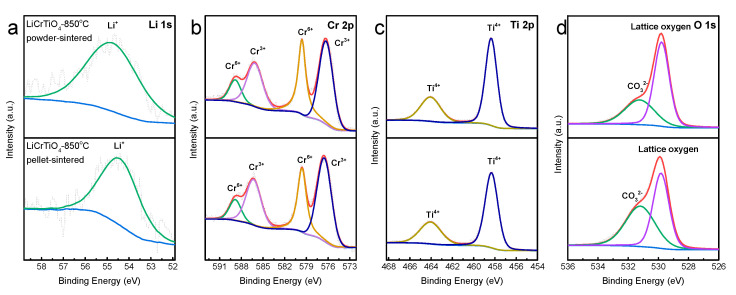
XPS core levels of (**a**) Li 1s; (**b**) Cr 2p; (**c**) Ti 2p, and (**d**) O 1s for LiCrTiO_4_ sintered at a temperature of 850 °C in a powder form (upper) and in a pellet form (lower).

The XPS measurement was adopted to analyze the difference between LiCrTiO_4_-850-powder and LiCrTiO_4_-850-pellet, as shown in Figure 9. The Li 1s peak at ca. 54.7 eV was observed for both samples, as shown in Figure 9a [32]. There are four binding energies: 576.4 eV for Cr^3+^ 2p_3/2_, 579.6 eV for Cr^6+^ 2p_3/2_, 586.3 eV for Cr^3+^ 2p_1/2_ and 588.9 eV for Cr^6+^ 2p_1/2_, as shown in Figure 9b [33,34,35]. The ratio between Cr^6+^ and Cr^3+^ is 0.735:1 for LiCrTiO_4_-850-powder and 0.632:1 for the LiCrTiO_4_-850-pellet, according to their peak areas. The Cr element exists in the form of Cr^3+^ in spinel LiCrTiO_4_, and the Cr^6+^ ions on the surface should be attributed to the high-temperature sintering in air atmosphere, especially for LiCrTiO_4_-850-powder. As shown in Figure 9c, the Ti 2p_3/2_ peak at 458.4 eV and Ti 2p_1/2_ peak at 464.1 eV indicate that the Ti element mainly exists in the form of Ti^4+^ [36]. As for the O 1s spectra in Figure 9d, the binding energies of 529.8 eV and 531.2 eV are assigned to the lattice oxygen and the CO_3_^2−^, respectively [37,38,39]. Here, the raw material of Li_2_CO_3_ might leave the CO_3_^2−^ on the particle surface, especially for LiCrTiO_4_-850-pellet. Therefore, the LiCrTiO_4_-850-powder has more Cr^6+^ and less CO_3_^2−^ on the particle surface than the LiCrTiO_4_-850-pellet, which should be correlative to the occurrence of electrochemical oscillation.

**Figure 10:**
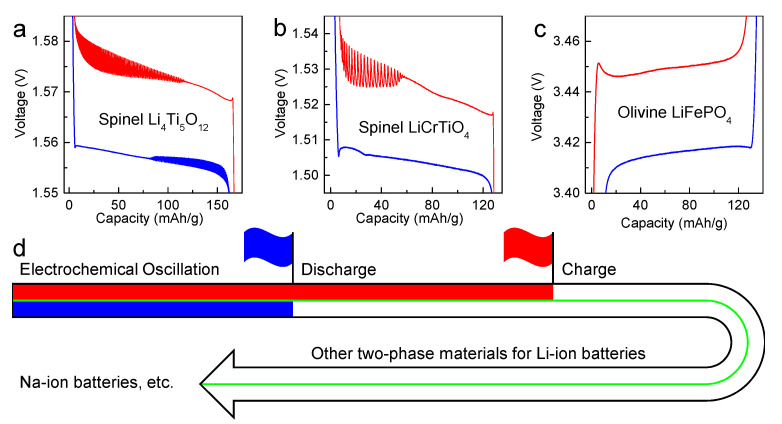
The galvanostatic discharge/charge curves of different electrode materials in Li-ion batteries: (**a**) Li_4_Ti_5_O_12_ prepared by carbothermic method; (**b**) LiCrTiO_4_ prepared by high-temperature solid-state method, and (**c**) commercial LiFePO_4_, (**d**) the research progress in electrochemical oscillation of Li-ion batteries.

Figure 10 shows the research progress in the electrochemical oscillation of Li-ion batteries, which was first discovered during the galvanostatic charge and discharge processes of spinel Li_4_Ti_5_O_12_ prepared by carbothermic method, as shown in Figure 10a. Here, our work reveals the electrochemical oscillation during the galvanostatic charge process of spinel LiCrTiO_4_, as shown in Figure 10b. Olivine LiFePO_4_ is a well-known two-phase cathode material in Li-ion batteries, but no electrochemical oscillation has been observed during the galvanostatic discharge and charge processes, as shown in Figure 10c, possibly owing to the anisotropic crystal structure and the poor electrical conductivity of LiFePO_4_. Thereby, the research of electrochemical oscillation has been expanded to the charge process of spinel LiCrTiO_4_, inspiring us to explore more two-phase electrode materials in Li-ion batteries, Na-ion batteries, etc., as shown in Figure 10d.

## 4. Conclusions

In this work, spinel LiCrTiO_4_ samples were synthesized by using a high-temperature solid-state method, characterized with SEM, XRD, Raman and XPS measurements, and electrochemically tested in Li-ion batteries to study the electrochemical oscillation. Through tailoring the sintering temperature, we found that the electrochemical oscillation during charging can be observed for the temperatures 800 to 900 °C, of which the range and amplitude increase with the temperature. Compared with the stoichiometric LiCrTiO_4_, the electrochemical oscillation is reduced in range and amplitude for the non-stoichiometric LiCrTiO_4_ samples, and it even disappears by reducing the Li content or increasing the Cr content. When the LiCrTiO_4_ is sintered in a pellet form, there is no electrochemical oscillation in the end of charging plateau. According to the XPS results, the powder-sintered LiCrTiO_4_ has more Cr^6+^ and less CO_3_^2−^ on the particle surface than the pellet-sintered LiCrTiO_4_, which should be correlative to the occurrence of electrochemical oscillation. Thereby, this work developed another two-phase material as the powder-sintered LiCrTiO_4_ to study the electrochemical oscillation, which would encourage us to explore more two-phase electrode materials in Li-ion batteries, Na-ion batteries, etc.

## Data Availability

The data presented in this study are available on request from the corresponding author.

## Supplementary Materials

The following are available online at https://www.mdpi.com/article/10.3390/ma14133624/s1, Supplementary Materials contained in Figure S1–S3, including enlarged XRD patterns of LiCrTiO_4_ for different sintering temperatures and different elemental ratios, SEM images of LiCrTiO_4_ composites with Li_2_Ti_3_O_7_, Li_4_Ti_5_O_12_ and Li_2_TiO_3_, respectively, sintered in a powder form at a temperature of 850 °C.
