# Supplementary material for: Electrochemical Oscillation during Galvanostatic Charging of LiCrTiO4 in Li-Ion Batteries
Source: Materials (Basel). 2021 Jun 29;14(13):3624. doi: 10.3390/ma14133624 (PMC8269718; doi:10.3390/ma14133624)
Supplement: Supplementary file 1 [file materials-14-03624-s001.zip › materials-1242653-supplementary.pdf]

materials

MDPI

Supporting Information

Electrochemical Oscillation during Galvanostatic Charging of $LiCrTiO_4$ in Li-ion Batteries

Zhijie Xu [1], Fangxu Hu[1], De Li [1,*] and Yong Chen [1,2,*]

[1] State Key Laboratory on Marine Resource Utilization in South China Sea, Hainan Provincial Key Laboratory of Research on Utilization of Si-Zr-Ti Resources, School of Materials Science and Engineering, Hainan University, Haikou 570228, China. 18085204210049@hainanu.edu.cn (Z.X.); 19085204210019@hainu.edu.cn (F.H.)
[2] Guangdong Key Laboratory for Hydrogen Energy Technologies; School of Materials Science and Hydrogen Energy, Foshan University, Foshan 528000, China.
* Correspondence: lidenju@sina.com (D.L.); ychen2002@163.com (Y.C.)

Supporting Information contains Figure S1–S3, including enlarged XRD (X-ray Diffraction) patterns of $LiCrTiO_4$ for different sintering temperatures and different elemental ratios, SEM (Scanning Electron Microscopy) images of $LiCrTiO_4$ composites with $Li_2Ti_3O_7$, $Li_4Ti_5O_{12}$ and Li_2TiO_3, respectively.

Citation: Xu, Z.; Hu, F.; Li, D.; Chen, Y. The Electrochemical Oscillation during the Galvanostatic Charging of $LiCrTiO_4$ in Li-ion Batteries. *Materials* **2021**, *14*, 3624. https://doi.org/10.3390/ma14133624

Academic Editor: Alberto Vertova, Digby D. Macdonald

Received: 16 May 2021
Accepted: 8 June 2021
Published: 29 June 2021

Publisher's Note: MDPI stays neutral with regard to jurisdictional claims in published maps and institutional affiliations.

Copyright: © 2021 by the authors. Submitted for possible open access publication under the terms and conditions of the Creative Commons Attribution (CC BY) license (http://creativecommons.org/licenses/by/4.0/).

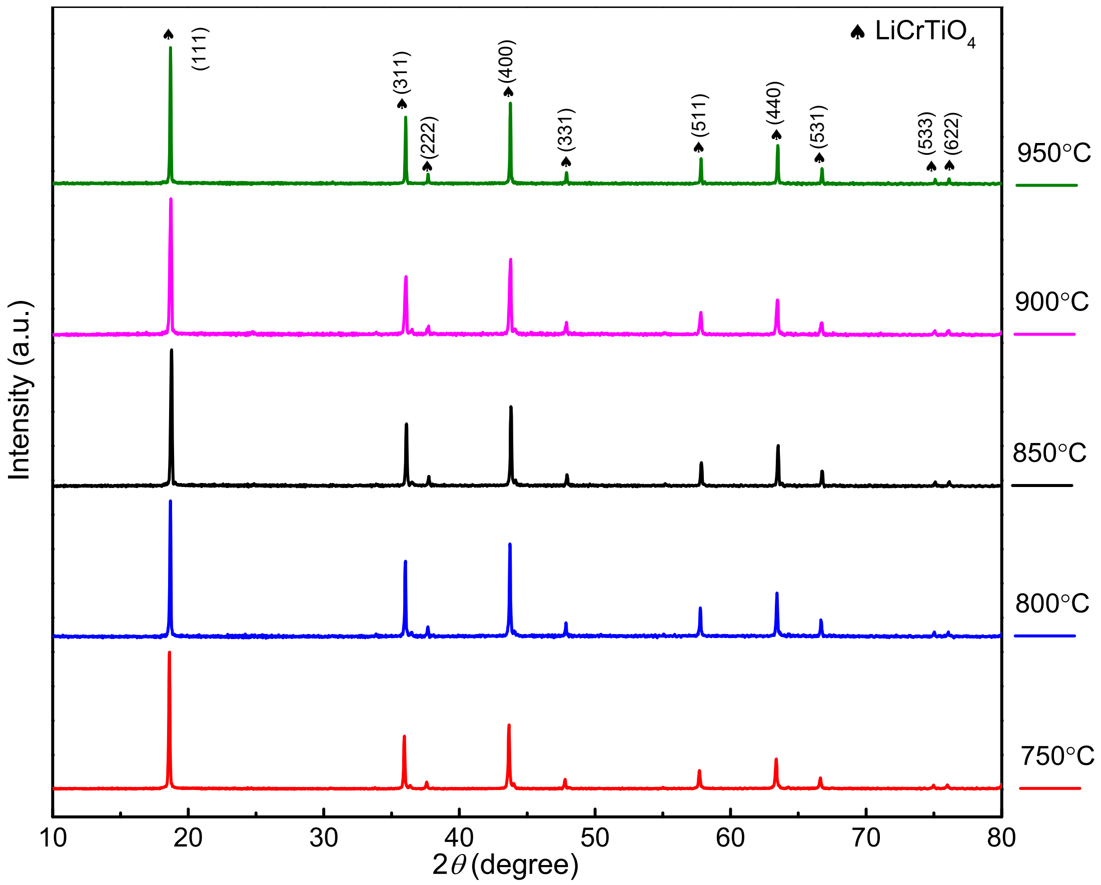

Figure S1. The enlarged XRD (X-ray Diffraction) patterns of $LiCrTiO_4$ sintered in a powder form at a temperature of 750 °C (red), 800 °C (blue), 850 °C (black), 900 °C (magenta) and 950 °C (olive).

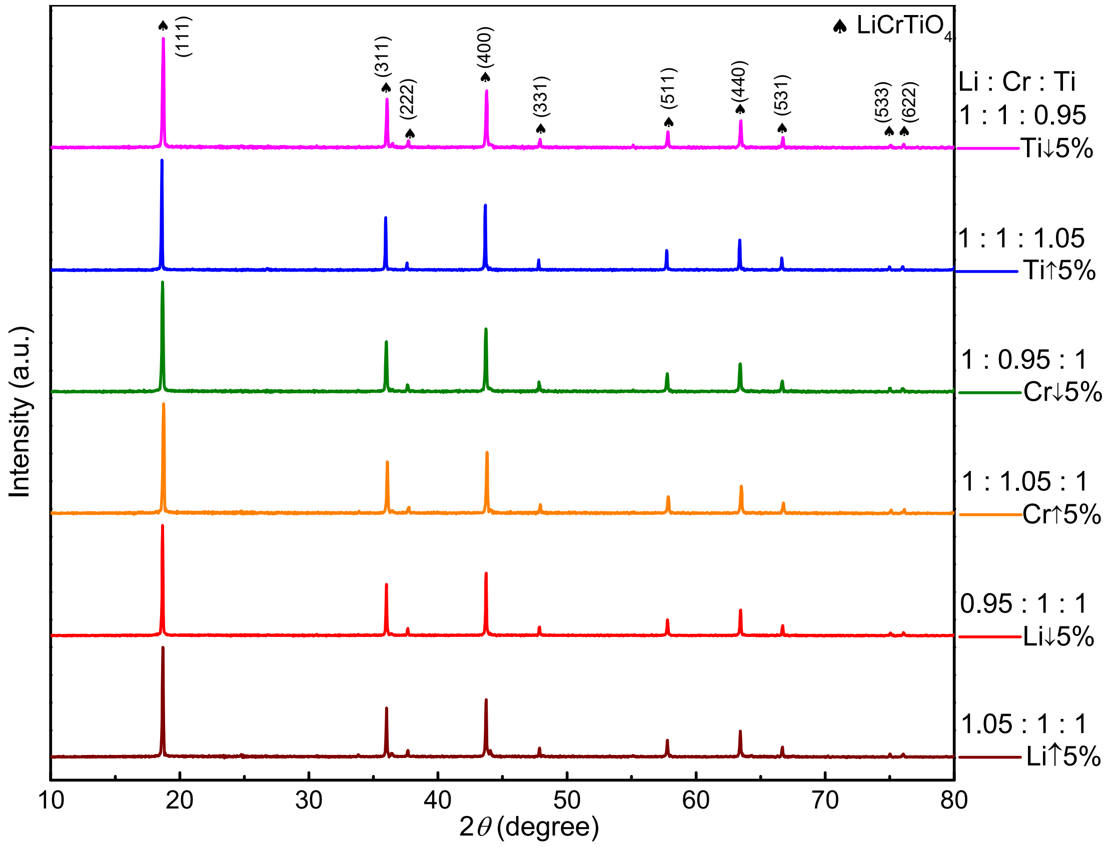

Figure S2. The enlarged XRD patterns sintered in a powder form at a temperature of 850 °C with an elemental ratio of Li:Cr:Ti: 1.05:1:1 (wine), 0.95:1:1 (red), 1:1.05:1 (orange), 1:0.95 (olive):1, 1:1:1.05 (blue) and 1:1:0.95 (magenta), respectively.

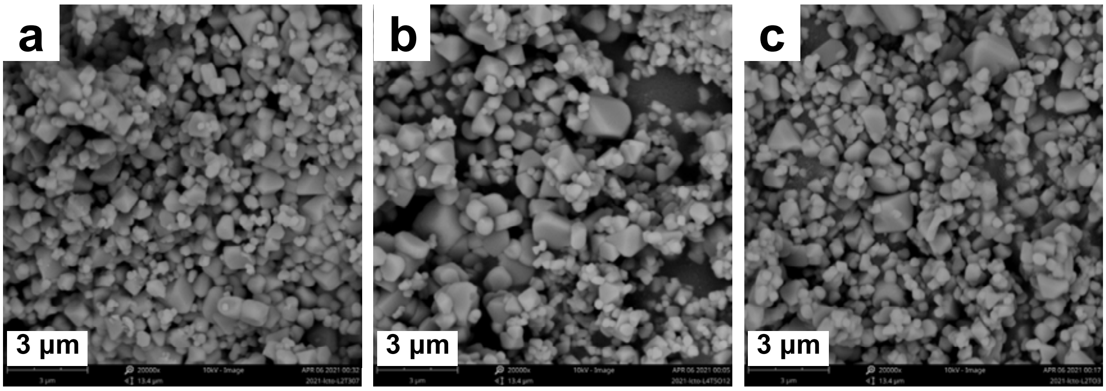

Figure S3. The SEM images of the composite as (**a**) $LiCrTiO_4$ + $\frac{1}{12}$ $Li_2Ti_3O_7$, (**b**) $LiCrTiO_4$ + $\frac{1}{20}$ $Li_4Ti_5O_{12}$ and (**c**) $LiCrTiO_4$ + $\frac{1}{4}$ Li_2TiO_3. Here, the raw materials Li_2CO_3 (Xilong Chemical Co., Ltd, Guangzhou, China) and TiO_2 (nano-sized, Aladdin, Shanghai, China) were weighted with an elemental ratio of Li:Ti as 2:3 for $Li_2Ti_3O_7$, 4:5 for $Li_4Ti_5O_{12}$ and 2:1 for Li_2TiO_3. The raw materials for $LiCrTiO_4$ were mixed with the raw materials for $Li_2Ti_3O_7$ ($Li_4Ti_5O_{12}$ or Li_2TiO_3) with a Ti-ion ratio of 4:1, which were sintered in a powder form at a temperature of 850 °C to obtain the composite of $LiCrTiO_4$ + $\frac{1}{12}$ $Li_2Ti_3O_7$ ($LiCrTiO_4$ + $\frac{1}{20}$ $Li_4Ti_5O_{12}$ or $LiCrTiO_4$ + $\frac{1}{4}$ Li_2TiO_3).
